# Specific Monoclonal Antibodies against African Swine Fever Virus Protease pS273R Revealed a Novel and Conserved Antigenic Epitope

**DOI:** 10.3390/ijms25168906

**Published:** 2024-08-15

**Authors:** Jiajia Zhang, Kaili Zhang, Shaohua Sun, Ping He, Dafu Deng, Pingping Zhang, Wanglong Zheng, Nanhua Chen, Jianzhong Zhu

**Affiliations:** 1 College Veterinary Medicine, Yangzhou University, Yangzhou 225009, China; 2 Joint International Research Laboratory of Agriculture and Agri-Product Safety, Yangzhou University, Yangzhou 225009, China; 3 Comparative Medicine Research Institute, Yangzhou University, Yangzhou 225009, China; 4 Jiangsu Co-Innovation Center for Prevention and Control of Important Animal Infectious Diseases and Zoonoses, Yangzhou University, Yangzhou 225009, China

**Keywords:** African swine fever virus, pS273R, monoclonal antibody, epitope, diagnosis

## Abstract

The African swine fever virus (ASFV) is a large enveloped DNA virus that causes a highly pathogenic hemorrhagic disease in both domestic pigs and wild boars. The ASFV genome contains a double-stranded DNA encoding more than 150 proteins. The ASFV possesses only one protease, pS273R, which is important for virion assembly and host immune evasion. Therefore, the specific monoclonal antibody (mAb) against pS273R is useful for ASFV research. Here, we generated two specific anti-pS273R mAbs named 2F3 and 3C2, both of which were successfully applied for ELISA, Western blotting, and immunofluorescence assays. Further, we showed that both 2F3 and 3C2 mAbs recognize a new epitope of N terminal 1–25 amino acids of pS273R protein, which is highly conserved across different ASFV strains including all genotype I and II strains. Based on the recognized epitope, an indirect ELISA was established and was effective in detecting antibodies during ASFV infection. To conclude, the specific pS273R mAbs and corresponding epitope identified will strongly promote ASFV serological diagnosis and vaccine research.

## 1. Introduction

African swine fever (ASF) is a highly contagious viral disease with a mortality rate approaching 100%, leading to significant economic losses to the swine industry worldwide [1,2]. ASF was first discovered in Kenya in 1921, identified in the sylvatic cycle, followed by its spread outside Africa mainly in European countries between 1957 and 1995 [3]. The ASF was introduced in Georgia in 2007 and spread to the Trans-Caucasus, Russian Federation, Eastern Europe, and the Baltic countries; into China in 2018; and spread to other Asia countries [4,5,6]. Because there is no effective and safe vaccine or antiviral drugs, ASF has drawn much public attention and scientific investigation [7,8,9].

The ASFV is a member of the *Asfarviridae* family, belonging to the nucleoplasmic large DNA virus (NCLDV) group, and it is the only known DNA arbovirus [10]. The ASFV genome contains a linear double-stranded DNA (170–190 kb) that encodes approximately 150 proteins [11,12]. ASFV gene *S273R* encodes a 31 kD protein, containing a “core domain” with conserved catalytic residues and exhibiting the characteristics of SUMO-1-specific cysteine proteases [13]. Two ASFV polyprotein precursors, pp220 and pp62, are cleaved by the intrinsic pS273R protease to produce p5, p34, p14, p37, and p150 (derived from pp220) and p8, p15, and p35 (derived from pp62) [14]. Thus, pS273R is involved in the viral late maturational step, which is essential for proper core assembly and infectivity of ASFV [15].

pS273R is not only essential for ASFV virion maturation but is also important in host immune evasion [16,17,18,19,20,21]. First, pS273R cleaves gasdermin D in a non-canonical way to inhibit inflammatory pyroptosis [21] and cleaves G3BP1 to antagonize the antiviral stress granule (SG) formation [17]. Second, pS273R evades host DNA sensing cGAS-STING pathway-mediated interferon (IFN) induction by disturbing the interactions between STING and IKKε [19], between TBK1 and IRF3 [16]. Additionally, pS273R was reported to mediate STAT2 degradation to inhibit IFN signaling [18] and mediate degradation of FoxJ1, a host antiviral factor [20].

However, in spite of the above pS273R biological functions and its crystal structure recently resolved [22], little is known about the antigenic characteristics of the pS273R protein. Further, the antigenic epitope of pS273R has not been described, which is necessary for the development of the detection method as well as the candidate vaccine. In this study, we expressed and purified the prokaryotic recombinant pS273R, and used it to immunize mice. Two monoclonal antibodies were generated and both recognized a conserved linear epitope at the N terminal 1–25 amino acids of pS273R. In addition, the epitope-based indirect ELISA is capable of specific detection of ASFV antibodies in clinical ASFV infection. These results not only provide the biological tools for ASFV research but also deepen our understanding of the antigenicity of ASFV protease.

## 2. Results

### 2.1. Production and Characterization of Recombinant pS273R Protein

**Figure 1:**
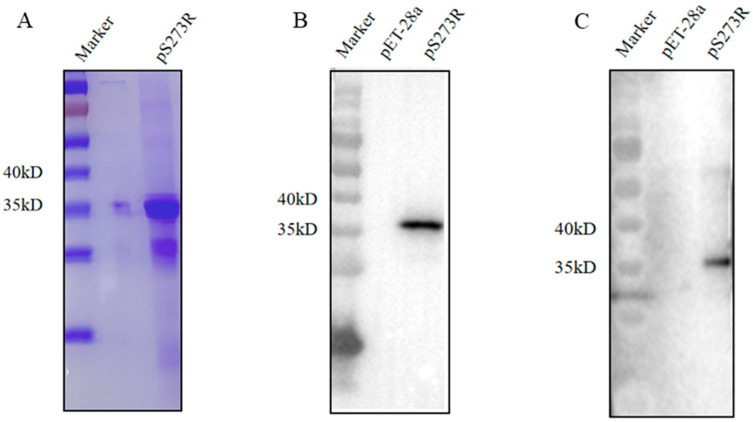
Production and identification of the pS273R recombinant protein. (**A**) The purified pS273R was verified by SDS-PAGE and Coomassie blue staining, with a major band of about 37 kD. (**B**,**C**) The purified pS273R protein was verified by Western blotting using the anti-His mAb (**B**) and the anti-ASFV positive pig serum (1:500). (**C**) The pET28a vector transformed bacterial protein was used as a control.

In this study, the ASFV pS273R coding sequence was cloned into the pET-28a vector and the recombinant plasmid was transformed into *E. coli* for protein expression. The recombinant protein was purified from the inclusion body and SDS-PAGE analysis showed that the purified pS273R had a high purity with a molecular mass of approximately 37 kD (Figure 1A). Further, Western blotting confirmed that the purified pS273R protein with an N terminal His tag was specifically recognized not only by anti-His mAb (Figure 1B) but also by anti-ASFV positive pig serum (Figure 1C), indicating the good antigenicity of the purified pS273R protein.

### 2.2. Generation of Monoclonal Antibodies (mAbs) against the pS273R Protein

**Figure 2:**
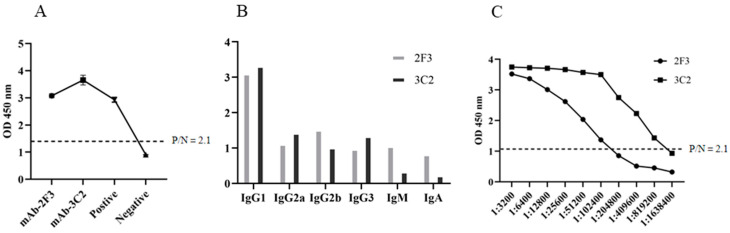
Preparation and characterization of anti-pS273R monoclonal antibodies. (**A**) The reactivity of mAbs was tested in pS273R protein-based indirect ELISA. The cell supernatants of hybridoma clones 2F3 and 3C2 were used as the primary antibodies, the SP2/0 cell supernatant was used as the negative control, and the serum of immunized mice was used as positive control. The dotted line denotes the P/N value of 2.1. (**B**) Subclasses of mAbs 2F3 and 3C2 were determined by the monoclonal antibody isotype identification kit from CELLWAY-LAB (Luoyang, China), according to the suggested protocol. (**C**) Measurement of the titers of ascite mAbs 2F3 and 3C2 by pS273R protein-based indirect ELISA. The dotted line denotes the P/N value of 2.1.

**Figure 3:**
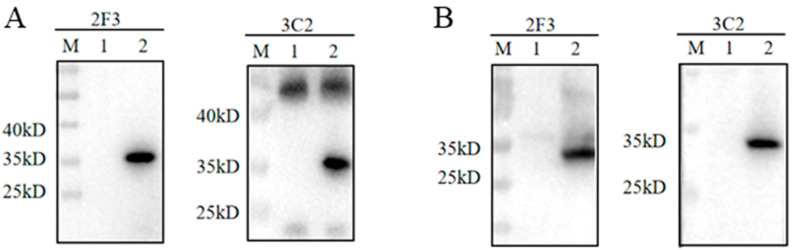
The specific reactivity of pS273R mAbs was analyzed by Western blotting. (**A**) 293T cells were transfected with pCAGGS-pS273R-2HA (lane 2) and pCAGGS vector control (lane 1). Cells were harvested and cell lysates were detected for exogenous pS273R by Western blotting with 2F3 and 3C2 ascite mAbs (1:1000) as primary antibodies. The 3C2 mAb but not 2F3 mAb recognized a non-specific ~45 kD band, suggesting that 2F3 mAb is more specific than 3C2. (**B**) Primary PAMs were infected with ASFV (MOI 0.1) (lane 2) and mock-infected (lane 1) for 96 h, and cell lysates were detected for endogenous pS273R by Western blotting with the ascite mAbs 2F3 and 3C2 (1:1000) as primary antibodies. The molecular weights of protein markers are indicated on the left. M, protein markers.

**Figure 4:**
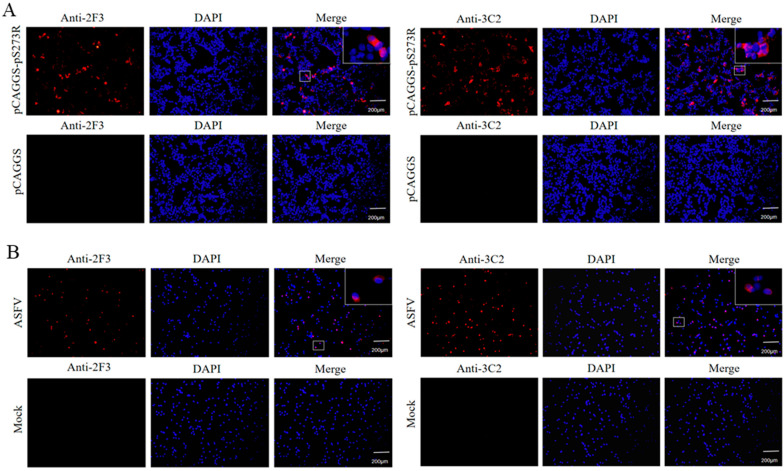
The specific reactivity of pS273R mAbs was analyzed with immunofluorescence. (**A**) 293T cells were transfected with the pCAGGS-pS273R and pCAGGS vector, respectively. Cells were fixed at 24 h post-transfection and stained with 2F3 or 3C2 ascite mAbs (1:200), together with Goat anti-mouse IgG H&L Alexa Fluor 594. Cellular nuclei were counterstained with 4′,6′-diamidino-2-phenylindole (DAPI). (**B**) Primary PAMs were infected with ASFV (MOI 0.1). Cells were fixed at 72 h post-infection and stained with ascite mAbs 2F3 or 3C2 (1:200), together with secondary antibody and DAPI. The boxed areas are magnified and placed on the upper-right corners of the merged images, clearly illustrating the main cytoplasmic localization of the pS273R protein.

The hybridomas were obtained by cell fusion and positive cell clones were selected by pS273R protein-based indirect ELISA [23]. After screening and subcloning three times, two hybridoma cell clones designated 2F3 and 3C2 were generated (Figure 2A). The mAbs produced by both 2F3 and 3C2 clones belong to the IgG1 subclass (Figure 2B). The ascites 2F3 and 3C2 mAbs were determined by ELISA to be 100,000–200,000 and 800,000–1600,000 in titers (Figure 2C), indicating the differential application values. We further analyzed the reactivity of these two ascite mAbs with the expressed proteins from 293T cells transfected with pCAGGS-S273R-HA (Figure 3A) and primary PAMs infected with ASFV (Figure 3B) by using Western blotting. The results showed that both 2F3 and 3C2 mAbs can specifically recognize exogenous pS273R (Figure 3A) and endogenous pS273R (Figure 3B), with the exogenous pS273R having a little higher molecular weight than endogenous pS273R. The results also suggested both mAbs recognize linear epitopes of pS273R. Similarly, the immunofluorescent assay demonstrated that both ascite mAbs are able to react with the expressed pS273R in transfected 293T cells (Figure 4A) as well as in ASFV-infected PAMs (Figure 4B), with the pS273R mainly localized in the cytoplasm. Together, the results indicated that produced mAbs are specific to ASFV pS273R.

### 2.3. Identification of the Precise Antigenic Epitope Recognized by Two pS273R mAbs

**Figure 5:**
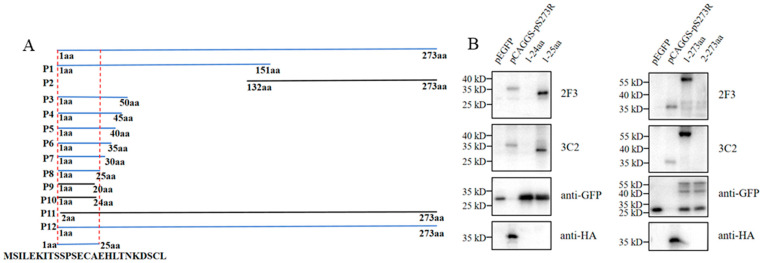
Identification of the antigenic epitope recognized by pS273R monoclonal antibodies. (**A**) Schematic of the strategy for mapping the epitope. The fragments with reactivity with pS273R mAbs are marked in blue, whereas those without reactivity with the mAbs are marked in black. (**B**) Western blotting analysis of the critical C terminal amino acid (left) and N terminal amino acid (right) for reactivity of pS273R fragments with the two ascite mAbs 2F3 and 3C2 (1:1000). P1 and P2 were cloned into the pCAGGS-HA vector, while P3–P12 were cloned in the pEGFP-N1 vector. Here the reactivity of P8 (1–25 aa), P10 (1–24 aa), and P11 (2–273 aa), P12 (1–273 aa) is shown, with the pCAGGS-pS273R-HA and pEGFP used as controls. The reactivities of the other fragments, P1–P7 and P9, are included in Figure S1. aa is an abbreviation for amino acid. The marks of molecular weights are indicated on the left.

As stated in Section 4 and illustrated in the schematic (Figure 5A), the initial testing with Western blotting demonstrated that both mAbs react with N fragment P1, not C fragment P2 (Figure 5A and Figure S1). Based on the results from P1, the progressive truncations of both N and C ends of P1 or full-length pS273R were performed and the resultant truncated fragments (P3–P12) were all tested for reactivity with pS273R mAbs by Western blotting (Figure S1). It turned out that N terminal amino acid 1 and C terminal amino acid 25 are critical for reactivity with both pS273R mAbs (Figure 5B). Clearly, both 2F3 and 3C2 mAbs recognize the linear epitope ^1^MSILEKITSSPSECAEHLTNKDSCL^25^.

### 2.4. Bioinformatics Analysis of the Identified Antigenic Epitope

**Figure 6:**
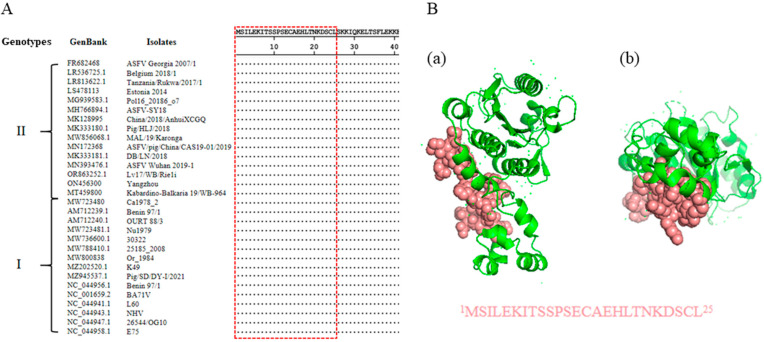
Conservation analysis of identified novel linear epitopes of the pS273R protein. (**A**) Alignment of the epitope (^1^MSILEKITSSPSECAEHLTNKDSCL^25^) in 30 representative genotype I and II ASFV strains. The red box indicates the identified conserved epitope. (**B**) Prediction of the pS273R structure by using PyMOL. The epitope recognized by two mAbs is displayed in pink, and shown in the front view (**a**) and bottom view (**b**), respectively.

In order to evaluate the conservation of the antigenic epitope across different ASFV strains, the pS273R protein sequences of 171 ASFV strains were downloaded from GenBank and aligned with the epitope sequence we identified. The alignment result showed that the epitope identified here is highly conserved across all ASFV strains except four genotype IX strains (Accession MH025918.1, MH025919.1, MH025920.1, and KM111295.1) (Table S1). Notably, the epitope sequences are identical in all genotype I and II ASFV strains, some of which are presented as representatives (Figure 6A). A 3D structure of ASFV pS273R (ID 6LJB) was obtained from the Protein Data Bank (PDB), and the visualization using PyMOL software revealed that the epitope is exposed on the surface of the pS273R protein in the 3D structure model, confirming its antigenicity (Figure 6B).

### 2.5. Establishment of Epitope-Based Indirect ELISA Detecting ASFV Antibody

**Figure 7:**
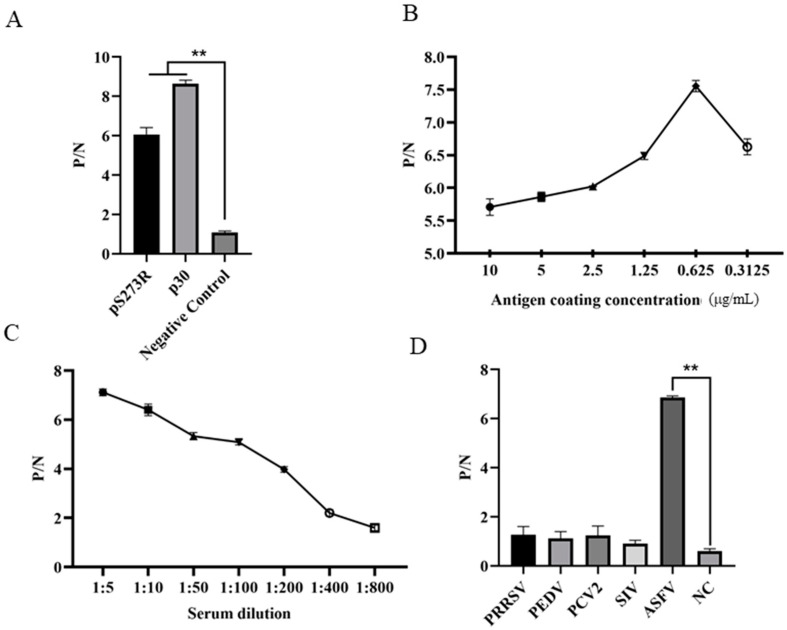
Establishment of indirect ELISA for detecting ASFV antibodies. (**A**) The pS273R epitope peptide, the positive p30 peptide (ETNECTSSFET), and a non-relevant control peptide (RSVPFEYYRIRKVKV) were used for coating at a concentration of 1 μg/mL (peptides were synthesized by GeneCreate Wuhan, China). The epitope-based ELISA was tested for detection of ASFV-positive serum, with a serum dilution of 1:10. The *p* values of pS273R vs. negative control and p30 vs. negative control are 0.002548 and 0.000347, respectively (n = 3). (**B**) The amount optimization of coating epitope peptide in indirect ELISA for detection of ASFV positive serum. (**C**) The optimization of serum dilution in peptide indirect ELISA with peptide coating concentration at 0.625 μg/mL. (**D**) Detection specificity of the established epitope-based indirect ELISA. PRRSV, PEDV, PCV2, SIV, and ASFV-positive porcine sera and negative porcine serum (NC) were used. The *p*-value of ASFV vs. NC is 0.000157 (n = 3). The signs ** in panels (**A**,**D**) denote *p* < 0.01 and are statistically very significant.

To verify the diagnostic feasibility of the pS273R mAb recognized epitope, we developed the epitope peptide-based indirect ELISA for the detection of ASFV antibodies. Preliminary testing was conducted with the coated pS273R epitope, plus a p30 epitope we identified and a non-relevant peptide as the controls, and the results showed that the pS273R and p30 epitope peptides, but not non-relevant peptide, were able to detect the antibody from an ASFV positive serum (Figure 7A). The pS273R epitope peptide was able to react specifically with the other two ASFV-positive sera (Figure S2A). Further optimization of the indirect ELISA determined that pS273R epitope peptide coated with the concentration of 0.625 μg/mL gives the highest P/N value in detecting ASFV-positive serum (Figure 7B). The dilution of ASFV-positive serum was determined to be 1:5, giving the highest P/N value (Figure 7C). The specificity of the epitope indirect ELISA was tested using different positive sera of porcine reproductive and respiratory syndrome virus (PRRSV), porcine epidemic diarrhea virus (PEDV), porcine circovirus 2 (PCV2), swine influenza virus (SIV), and ASFV. The results showed that only the ASFV-positive serum, but not others, gives a positive P/N value (Figure 7D). The cut-off values of peptide-based ELISA from the detection of 30 negative porcine sera were determined to be 0.415–0.512, with OD_450_ < 0.415 as negative and OD_450_ > 0.512 as positive (Figure S2B), whereas the cut-off values of pS273R protein-based ELISA were 0.361–0.444 (Figure S2C). Therefore, our preliminary results suggested that the pS273R mAb recognized epitope can be applied for immunological detection of ASFV infection.

## 3. Discussion

ASF is a devastating infectious disease in pigs, severely threatening the global pig industry [1]. The World Organization for Animal Health (WOAH) takes ASF as a notifiable disease [2]. Yet, currently, there is still no commercial vaccine and effective therapeutics, and improving the level of biosafety is still the main means to prevent and control ASF [24]. Due to its genetic complexity, many ASFV genes are still poorly studied, which may contribute to the insufficient understanding of its pathogenesis and hinder the progress of ASFV vaccine development and disease control [25]. According to previous investigations, the ASFV-only protease pS273R plays an important role in ASFV infection and host immune evasion [13,14,15,16,17,18,19,20,21]. Accordingly, mAb against ASFV pS273R may serve as a useful tool for viral research. Here we generated two specific mAbs against pS273R, which can specifically detect ASFV in various immunological assays including ELISA, Western blotting, and immunofluorescence (Figure 2, Figure 3 and Figure 4), exhibiting great value in diagnosis.

In order to obtain more complete mAbs and the corresponding antigenic epitopes of pS273R, we used the full-length pS273R protein to immunize mice for subsequent cell fusion. The full-length pS273R is a good antigenic since it is recognized by ASFV-positive pig serum in Western blotting (Figure 1C). Although only two mAbs were obtained, both mAbs recognize one identical linear B-cell epitope (aa 1–25) (Figure 5), indicating the predominance of this antigenic epitope. As far as we know, it is the first reported ASFV pS273R antigenic epitope. As a protease, pS273R is composed of an N-terminal arm domain (aa 1–83) and C terminal core domain (aa 84–273), with the arm domain playing an important role in maintaining the enzyme activity of the core domain [22]. As shown in the same study [22], the N terminal 1–20 amino acids are also necessary for the protease activity of pS273R. Our mAbs recognize the aa 1–25; however, whether these mAbs can block the pS273R protease activity is unknown and it deserves investigation. Among 150 plus ASFV proteins, there are about 50 viral structural proteins [11]. The generation of mAbs and identification of antigenic epitopes have been reported for several common viral structure proteins, such as p72 [26,27,28,29,30,31], p54 [32,33,34,35,36,37], p30 [38,39,40,41,42,43], and p17 [44]. Even though pS273R is a protease, it exists in the core-shell of ASFV virions together with the polyproteins [13]. Therefore, it is reasonable that pS273R exhibits antigenicity during ASFV infection (Figure 1C) and the epitope recognized by its mAb is able to detect antibodies in ASFV-positive serum.

ASFV was first discovered in Kenya in 1921 and there have been 24 genotypes in Africa [1,45]. Between 1957 and 1995, genotype I ASFV emerged in Europe, Russia, the Caribbean, and South America [3]. In 2007, genotype II ASFV emerged in the Republic of Georgia and continued to spread through the Caucasus region and subsequently into the Russian Federation and Eastern Europe, where it has continued to circulate and spread [10]. In August 2018, the first ASF case was reported in China and genotype II ASFV was identified [5]. Until now, genotype II ASFV strains are mainly epidemic strains in China, whereas genotype I ASFV also emerged in domestic pigs and caused chronic infection in China [46,47]. Therefore, the diagnosis of genotype I and/or genotype II ASFV infections is particularly important. The antigenic epitope recognized by pS273R mAbs we generated is highly conservative across different ASFV strains and is identical to all genotype I and genotype II ASFV strains (Figure 6). Thus, the mAbs and antigenic epitope can be used for general detection of genotype I and genotype II ASFV infections.

In this study, we developed two indirect ELISAs for the detection of ASFV antibodies by using the pS273R protein and antigenic epitope (Figure 7), respectively. Both ELISAs were able to detect ASFV pS273R antibody and the epitope-based ELISA was supposed to be more specific than the pS273R-based ELISA due to the higher purity of synthesized antigenic peptide. Indeed, we observed a higher positive cut-off value in epitope-based ELISA than in pS273R-based ELISA (Figure S2B,C). Additionally, the pS273R mAbs we developed can also be used alone or in combination in developing competitive ELISA for the detection of ASFV antibodies. However, we do not know which ELISA will be better for the detection of ASFV antibodies in clinical samples. To this end, the three types of ELISA need to be tested side by side with enough clinical serum samples, which deserve to be further investigated. Together, our results suggest that the ASFV pS273R, its mAbs, and the corresponding epitope facilitate the development of serological diagnosis and ASF prevention and control.

In summary, we generated two specific mAbs against ASFV pS273R protease, identified a highly conserved linear B-cell epitope of pS273R, and established the indirect ELISA for the detection of ASFV antibodies. These results contribute to the study on ASFV pS273R function and the development of ASFV diagnostic and vaccine candidates.

## 4. Materials and Methods

### 4.1. Mice, Cells, Virus, and Sera

The 6- to 8-week-old, female, BALB/c mice were purchased from the Laboratory Animal Center of Yangzhou University. HEK293T cells (293T) and myeloma cell line SP2/0 were cultured in Dulbecco modified Eagle medium (DMEM, Hyclone Laboratories, Logan, UT, USA) supplemented with 100 IU/mL of penicillin plus 100 μg/mL streptomycin and 10% fetal bovine serum (FBS) (Gibco, Grand Island, NY, USA). Primary porcine alveolar macrophages (PAMs) were obtained from 2-month-old domestic pigs by regular bronchoalveolar lavage, and incubated in RPMI 1640 medium (Hyclone Laboratories) containing 100 IU/mL of penicillin plus 100 μg/mL streptomycin and 10% FBS. Cells were grown at 37 °C in a 5% CO_2_ humidified incubator. Cell transfections were performed with the reagents Lipofectamine 2000 and Opti-MEM (ThermoFisher Scientific, Shanghai, China), according to the suggested procedure. The ASFV strain (genotype II, GenBank accession ON456300) was preserved in the animal biosafety level 3 (ABSL-3) of Yangzhou University approved by the Ministry of Agriculture and Rural Affairs (07140020201109-1) and the PAMs were infected with 0.1 MOI ASFV for 72 h. The positive porcine sera of PRRSV, PEDV, PCV2, SIV, and ASFV, and negative porcine sera were stored in our lab. The animal experiment was in strict accordance with the Guidance for the Care and Use of Laboratory Animals of Yangzhou University (SYXK(JS)-2021-0026).

### 4.2. Expression and Purification of pS273R Protein

The ASFV pS273R gene coding sequence containing stop codon was PCR amplified from pCAGGS-S273R-HA constructed in our laboratory and cloned into *Nde*I and *Xho*I sites of pET-28a vector by Seemless/In-Fusion Cloning (2 × MultiF Seamless Assembly Mix, Abclonal, Wuhan, China). The recombinant plasmid amplified in *E. coli* DH5α cells was transformed into *E. coli* BL21/DE3 competent cells and treated with 1 mM isopropyl-β-d-1-thiogalactoside (IPTG) at 16 °C for 14 h to induce pS273R expression. The bacteria pellet was collected by centrifugation, resuspended in phosphate-buffered saline (PBS), and sonicated (Biosafer, Nanjing, China) on ice. After centrifugation, the supernatant was removed, and the pelleted inclusion bodies were resuspended in 8 mM urea buffer and were gently shaken at 4 °C overnight, followed by gradient dialysis (6, 4, 2, 0 mM urea buffer) and subsequent refolding [23]. The purified protein was confirmed by Western blotting with His mAb (TransGen Biotech, Beijing, China).

### 4.3. Generation of Anti-pS273R Monoclonal Antibodies (mAbs)

BALB/c mice were first immunized with 100 μg purified pS273R protein emulsified in complete Freund’s adjuvant at a 1:1 ratio, and then with purified pS273R emulsified in incomplete Freund’s adjuvant (twice) at 2-week intervals. Three days after the last immunization, tail vein blood was taken, and serum pS273R antibody was determined by indirect ELISA. The mouse presenting the highest antibody titer was chosen for the final boost without adjuvant and subsequently sacrificed three days later for cell fusion with SP2/0 cells. The hybridomas that secreted pS273R-specific antibodies were screened by pS273R protein-based indirect ELISA and confirmed by Western blotting. Positive hybridomas were subcloned three times by limiting dilution and tested again for antibody production. Subsequently, the mouse ascites were made from the two positive hybridomas.

### 4.4. Identification of the Anti-pS273R mAb Isotypes/Subclasses

The isotypes/subclasses of pS273R mAbs were determined by the mouse mAb isotype identification kit (Cat No, C060101, CELLWAY-LAB, Louyang, China) according to the manufacturer’s instructions. Briefly, 50 μL culture supernatant from each hybridoma cell clone or 50 μL each mAb ascite fluid mixed with 50 μL sample diluent from the kit was dispensed to the detection plate wells, with each mAb 6 replicate wells (100 μL/well). After incubating at 37 °C for 30 min, the plate was washed 5 times and the 6 types of enzyme-linked secondary antibodies were added into the 6 wells of all mAb samples, accordingly (100 μL/well). After incubating at 37 °C for 30 min, the plate was washed 5 times, and the substrates A and B from the kit were added (50 μL each) into all the wells and the chromogenic reaction was developed at 37 °C for 20 min in the dark. The reaction was stopped by the stopping solution (50 μL/well) and the optical density at 450 nm (OD_450_) was measured by a spectrophotometer (ALL-SHENG, Hangzhou, China). The mAb isotype was determined based on the higher OD_450_ value of the exact type of 6 secondary antibodies.

### 4.5. Mapping of the Linear B-Cell Epitope of pS273R Protein

First, the pS273R sequence was separated into two fragments for cloning and expression to test the reactivity with pS273R mAbs. Next, based on the reacted fragment, progressive truncations were performed at both the N and C terminal ends of the fragment or full-length pS273R. The reactivity of different truncated fragments with pS273R mAbs was tested, and the critical amino acids at both N and C ends for reactivity with pS273R mAbs were determined, from which the minimal epitope was deduced. Specifically, 12 pS273R fragments (P1–P12) were designed and used, with all cloning PCR primers listed in Table S2. All the pS273R fragments were PCR amplified, P1 and P2 were cloned into the *EcoR*I/*EcoR*V sites of pCAGGS-HA vector, and P3–P12 were cloned into the *Bgl*II/*Kpn*I sites of pEGFP-N1 vector by Seemless/In-Fusion Cloning with 2× MultiF Seamless Assembly Mix. The recombinant plasmids were transfected into 293T cells for protein expressions, and the reactivity of different truncated fragments in transfected 293 T cells with pS273R ascite mAbs (1:1000) was tested by using Western blotting.

### 4.6. Western Blotting

The reactivity of anti-His mAb with the recombinant pS273R protein and anti-pS273R mAb with pS273R in transfected 293T cells and ASFV-infected PAMs was evaluated using Western blotting. Specifically, the cells were lysed in RIPA buffer (50 mM Tris pH 7.2, 150 mM NaCl, 1% sodium deoxycholate, 1% Triton X-100), and the extracted proteins were separated by 6–10% SDS-polyacrylamide gels and transferred to PVDF membranes. Membranes were blocked for 1 h using 5% non-fat dry milk Tris-buffered saline, with 0.1% Tween-20 (TBST). Then, the membrane was incubated with the primary mAbs (1:1000 anti-HA, and 1:1000 pS273R ascite mAbs) in a blocking buffer at 4 °C overnight. Next, the membrane was incubated with HRP-conjugated Goat Anti-Mouse IgG (1:10,000, BBI, Shanghai, China) for 1 h. Upon addition of enhanced chemiluminescence (ECL) substrate (Tanon, Shanghai, China), the protein signals were visualized and captured by the Western blot imaging system (Tanon).

### 4.7. Immunofluorescence Assay

293T cells were transfected with pCAGGS-S273R-HA for 24 h, whereas primary PAMs were infected with ASFV at a multiplicity of infection (MOI) of 0.1 for 72 h. Subsequently, the cells were fixed in 4% paraformaldehyde for 30 min, permeabilized by 0.5% Triton X-100, and then blocked with 5% BSA. The treated cells were incubated with pS273R mAbs (1:200 ascites) overnight, and then Goat anti-mouse IgG H&L Alexa Fluor 594 (1:500, Abcam, Shanghai, China) for 1 h, followed by DAPI staining (0.5 μg/mL, Beyotime, Shanghai, China) for 15 min. The stained cells were visualized under a fluorescence microscope (Olympus, Tokyo, Japan).

### 4.8. Enzyme-Linked Immunosorbent Assay (ELISA)

For pS273R protein-mediated indirect ELISA, the ELISA plate wells were coated with pS273R purified protein diluted in PBS at a final concentration of 0.625 μg/mL at 4 °C overnight, followed by washing and blocking with 5% skim milk at 37 °C for 2 h. Hybridoma supernatants were added to each well (100 μL/well) and incubated for 2 h at 37 °C. The SP2/0 cell supernatant was used as the negative control. After washing with PBST, the secondary antibody Goat anti-Mouse IgG-HRP (1:10,000 dilution, TransGen Biotech, Beijing, China) was added (100 μL/well) and incubated at 37 °C for 1 h. Next, 3,3′,5,5′-tetramethylbenzidine (TMB) was added (50 μL/well) and incubated for 15 min at 37 °C in the dark. The reaction was stopped with 0.5 M H_2_SO_4_ and the optical density at 450 nm (OD_450_) was measured by a spectrophotometer (ALLSHENG, Hangzhou, China). The ratios of hybridoma supernatants to negative supernatant (P/N) were calculated, with P/N ≥ 2.1 as positive.

For epitope-mediated indirect ELISA, ELISA plate wells were coated with epitope peptide diluted with PBS at the concentration of 0.3125–10 μg/mL at 4 °C overnight, followed by washing and blocking with 5% BSA at 37 °C for 2 h. Diluted positive porcine sera (1:5–1:800, 100 μL/well) were added and incubated at 37 °C for 2 h. Normal healthy pig serum was used as the negative control. After washing with PBST, secondary antibody Goat anti-Swine IgG-HRP (1:10,000, Proteintech, Wuhan, China) was added (100 μL/well) and incubated at 37 °C for 1 h, followed by the addition of substrate TMB, stopping with 0.5 M H_2_SO_4_ and OD_450_ measurement. The ratios of positive sera to negative serum (P/N) were calculated, with P/N ≥ 2.1 as positive.

### 4.9. The Titration of Anti-pS273R Ascite mAbs

The titers of pS273R ascite mAbs were measured by the above pS273R protein-mediated indirect ELISA. The 2-fold serially diluted ascite mAbs 2F3 and 3C2 (1:3200 to 1:1,638,400) were added (100 μL/well) as the primary antibodies in the indirect ELISA, whereas the diluent was used as the negative control. The secondary antibody Goat anti-mouse IgG-HRP was added followed by TMB incubation. The reaction was stopped with 0.5 M H_2_SO_4_ and the OD_450_ was measured. The P/N was calculated based on the average OD_450_ values measured (n = 3). The maximal dilutions of ascite mAbs with the P/N ≥ 2.1 were regarded as the mAb titers.

### 4.10. Bioinformatics Analysis

To verify the conservation of the identified pS273R epitope, the pS273 protein sequences of 171 ASFV strains from GenBank were downloaded. The amino acid sequence alignment and conservation analysis was implemented by Clustal W in MegAlign software, version 7.1.0 (DNAstar, Madison, WI, USA). The spatial distribution and the structure of the identified epitope within pS273R (PDB: 6LJB) were visualized by the PyMOL Molecular Graphics System (Version 2.4.0, Schrödinger, LLC, New York, NY, USA).

### 4.11. Statistical Analysis

All the experiments were repeated at least three times and the representatives were presented. The results were analyzed by using GraphPad Prism 8.2 software (Graph Pad Prism Inc., Boston, MA, USA). Data were presented as the mean ± SD of three replicates. Statistical difference between samples was assessed using Student’s *t*-test. *p* < 0.05 was set as the threshold for statistical significance.

## Data Availability

The authors confirm that the data supporting the findings of this study are available within the article and its Supplementary Materials.

## Supplementary Materials

The following supporting information can be downloaded at: https://www.mdpi.com/article/10.3390/ijms25168906/s1.
