# Supplementary material for: Specific Monoclonal Antibodies against African Swine Fever Virus Protease pS273R Revealed a Novel and Conserved Antigenic Epitope
Source: Int J Mol Sci. 2024 Aug 15;25(16):8906. doi: 10.3390/ijms25168906 (PMC11354548; doi:10.3390/ijms25168906)
Supplement: Supplementary file 1 [file ijms-25-08906-s001.zip › Supplementary Figures.pdf]

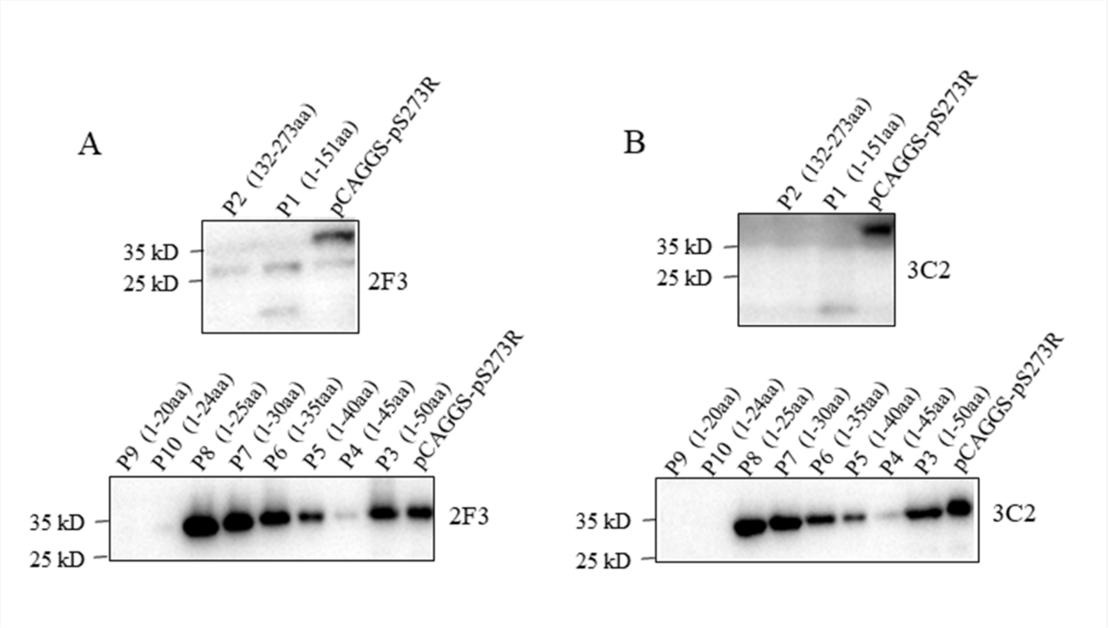

Fig S1. Identification of the antigenic epitope recognized by pS273R monoclonal antibodies. (A) The reactivity of ascite mAb 2F3 with HA tagged P1 and P2 (top), and GFP tagged P3-P10 (low) in Western blotting. (B) The reactivity of ascite mAb 3C2 with HA tagged P1 and P2 (top), and GFP tagged P3-P10 (low) in Western blotting.

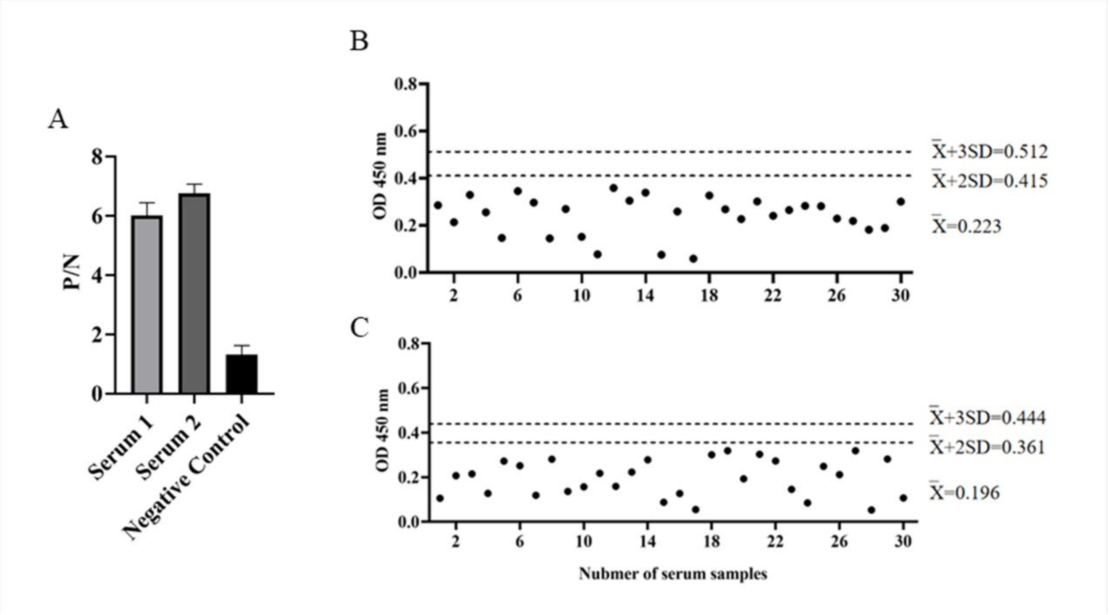

Fig S2. The reactivity of antigenic peptide with ASF positive and negative sera in ELISA. (A) The reactivity of peptide with two ASF positive sera. The negative serum was used as negative control. (B and C) The measurement of cut off values based on 30 clinical negative sera by the optimized peptide based ELISA (B) and pS273R based ELISA (C). The $\overline{X}$ denotes average values, and SD denotes standard error. The calculated cut off values of peptide ELISA are 0.415-0.512, with $OD_{450}<0.415$ as negative and $OD_{450}>0.512$ as positive.
