# Supplementary material for: Specific Monoclonal Antibodies against African Swine Fever Virus Protease pS273R Revealed a Novel and Conserved Antigenic Epitope
Source: Int J Mol Sci. 2024 Aug 15;25(16):8906. doi: 10.3390/ijms25168906 (PMC11354548; doi:10.3390/ijms25168906)
Supplement: Supplementary file 1 [file ijms-25-08906-s001.zip › Table S2.pdf]

Table S2. The cloning PCR primers used in this study

pS273R fragments	Sequences (5' - 3')
pCAGGS-pS273R-2HA	F:TGTCTCATCATTTTGGCAAA*GAATTC*ATGTCTATATTAGAAA<br>R:ATCGTATGGGTAGCTGGT*GATATC*TGCGATGCGAAACAGATG
pET28a-pS273R	F:GGTGCCGCGCGGCAGC*CATATG*ATGTCTATATTAGAAAAAAT<br>R:GTGGTGGTGGTG*CTCGAG*TTATTATGCGATGCGAAACAGATG
P1	F: CATTTTGGCAAA*GAATTC*ATGTCTATATTAGAAAAAAT<br>R: TATGGGTAGCTGGT*GATATC*GTTACAAGGACGCTTGAC
P2	F: CATTTTGGCAAA*GAATTC*GTATATAAGGGAGAAGAGCT<br>R: TATGGGTAGCTGGT*GATATC*TGCGATGCGAAACAGATG
P3	F:gcgctaccggactc*agatct*ATGTCTATATTAGAAAA<br>R:atcccgggcccgc*ggtacc*gtGCAGGACTCCGAATCG
P4	F:gcgctaccggactc*agatct*ATGTCTATATTAGAAAA<br>R:atcccgggcccgc*ggtacc*gtGCAACCGAGTGTCTCTT
P5	F:gcgctaccggactc*agatct*ATGTCTATATTAGAAAA<br>R:atcccgggcccgc*ggtacc*gtTTTTTTTTCCAAAAAAG
P6	F:gcgctaccggactc*agatct*ATGTCTATATTAGAAAA<br>R:atcccgggcccgc*ggtacc*gtAGAGGTGAGCTCTTTT
P7	F:gcgctaccggactc*agatct*ATGTCTATATTAGAAAA<br>R:atcccgggcccgc*ggtacc*gtTTGTATTTTTTTACTTA
P8	F:gcgctaccggactc*agatct*ATGTCTATATTAGAAAA<br>R:atcccgggcccgc*ggtacc*gtTAAACAGCTATCTTTGT
P9	F:gcgctaccggactc*agatct*ATGTCTATATTAGAAAA<br>R:atcccgggcccgc*ggtacc*gtGTTTGTAAGATGCTCT
P10	F:gcgctaccggactc*agatct*ATGTCTATATTAGAAAA<br>R:atcccgggcccgc*ggtacc*gtACAGCTATCTTTGTTT
P11	F:gcgctaccggactc*agatct*TCTATATTAGAAAAAAT<br>R:atcccgggcccgc*ggtacc*gtTGCGATGCGAAACAGA
P12	F:gcgctaccggactc*agatct*ATGTCTATATTAGAAAA<br>R:atcccgggcccgc*ggtacc*gtTGCGATGCGAAACAGA

Note: All the amplified PCR fragments were ligated into vectors by Seamless Cloning/In-Fusion Cloning. F, forward; R, reverse. The restriction sites are italic.
