# Supplementary figures and images for: Specific Monoclonal Antibodies against African Swine Fever Virus Protease pS273R Revealed a Novel and Conserved Antigenic Epitope
Source: Int J Mol Sci. 2024 Aug 15;25(16):8906. doi: 10.3390/ijms25168906 (PMC11354548; doi:10.3390/ijms25168906)

Figure 1

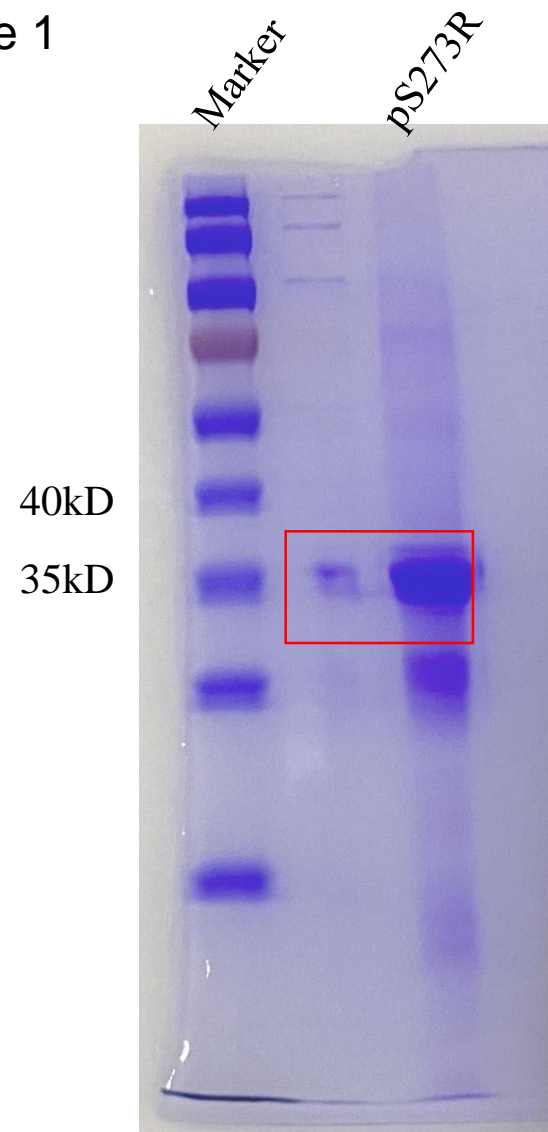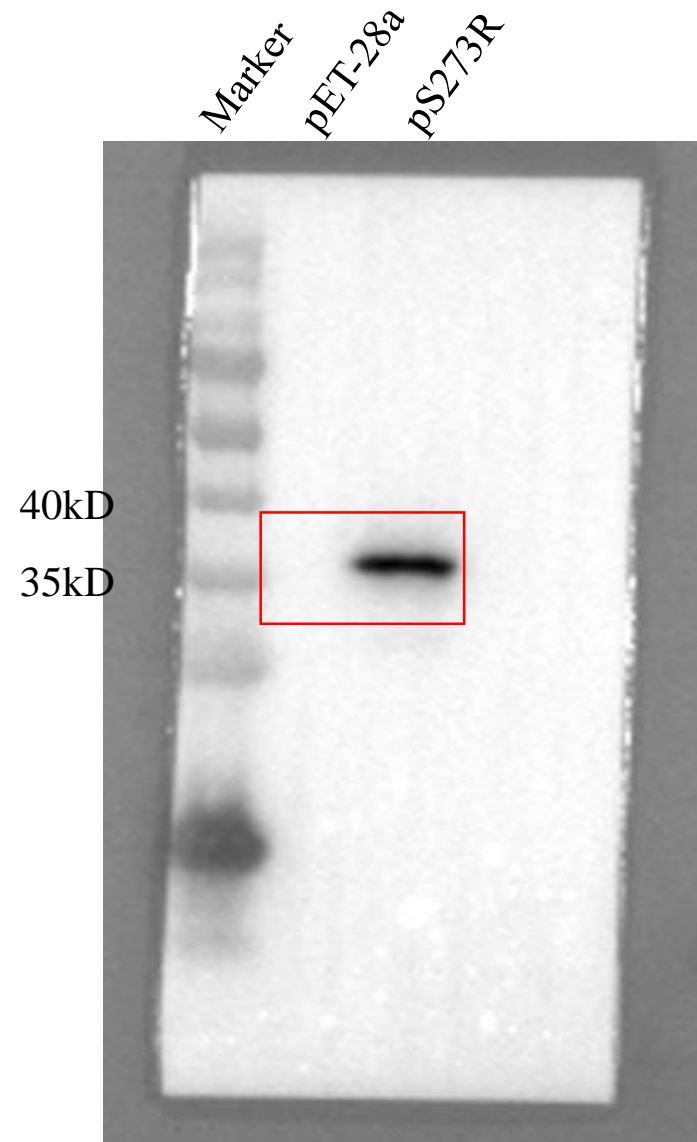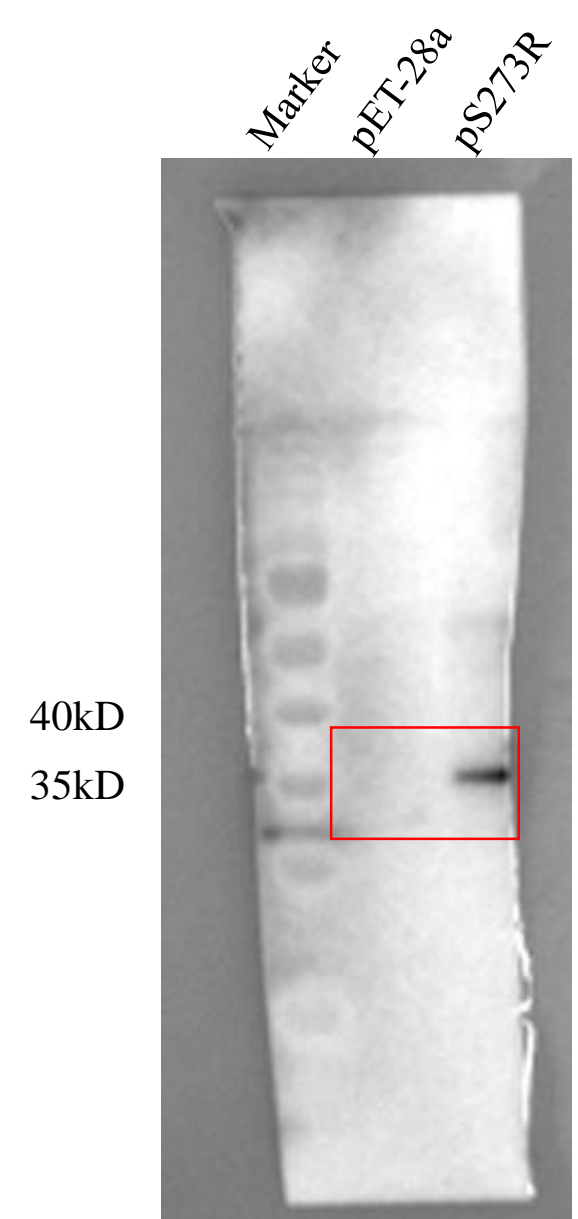

Figure 3

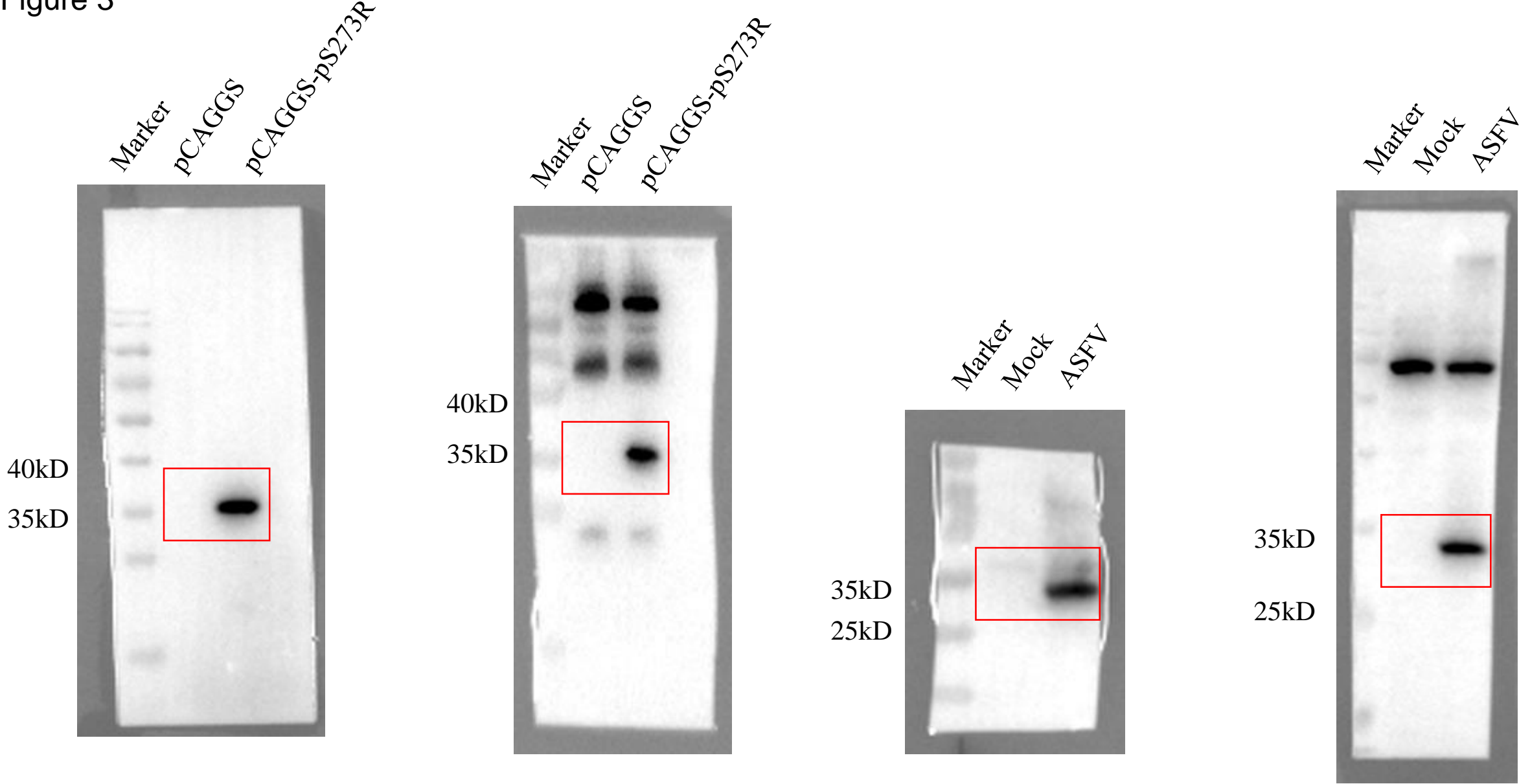

Figure 5

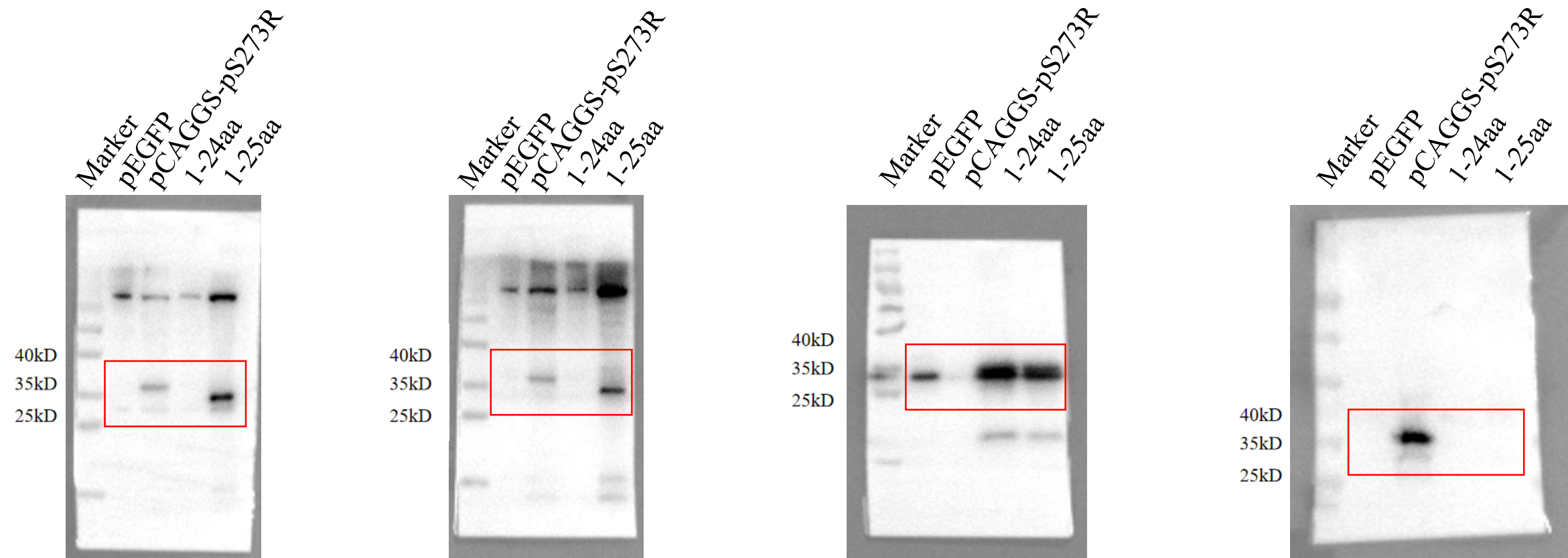

Figure 5

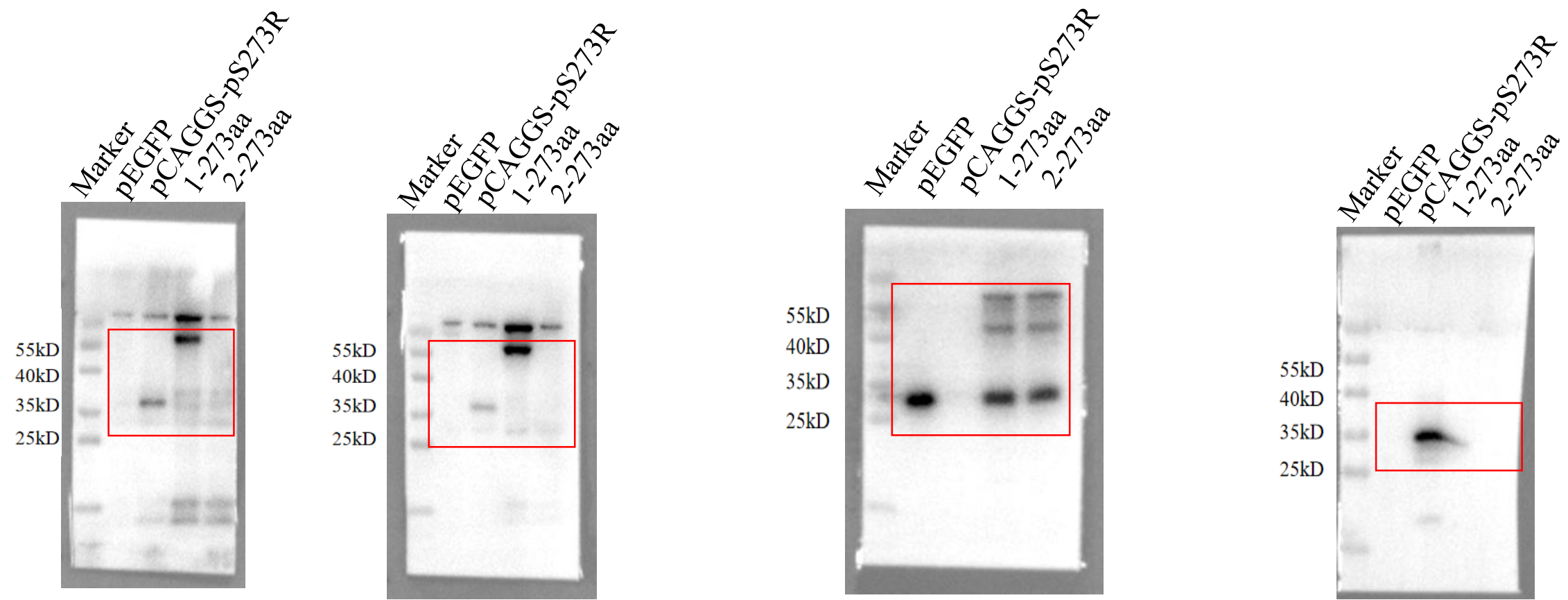

Figure S1

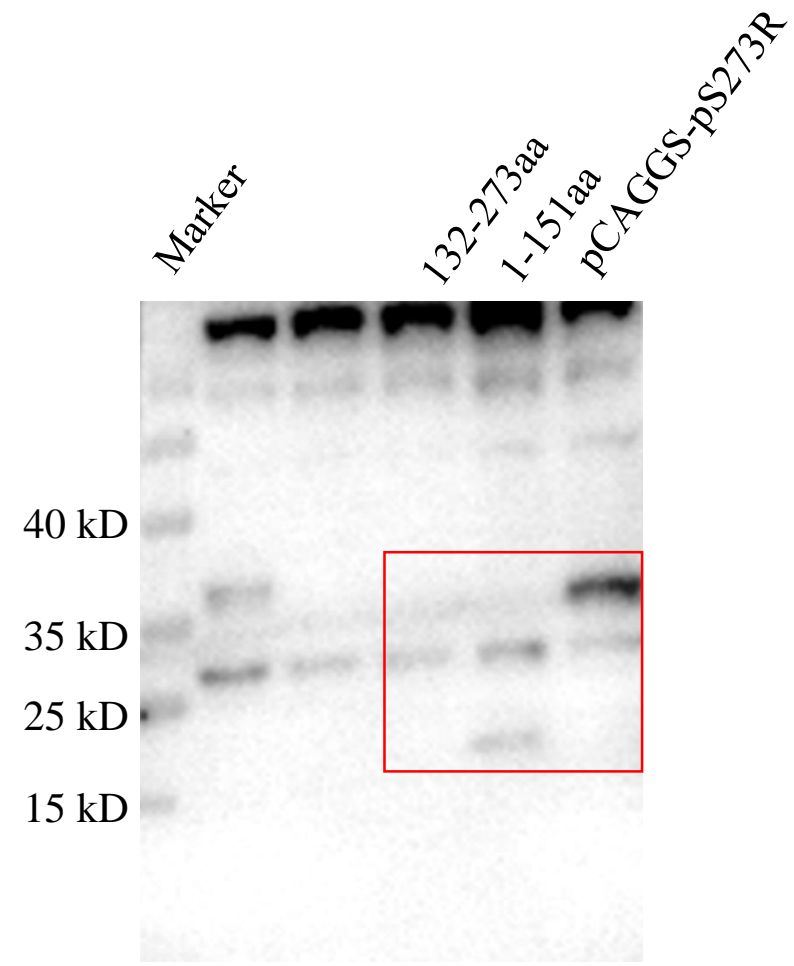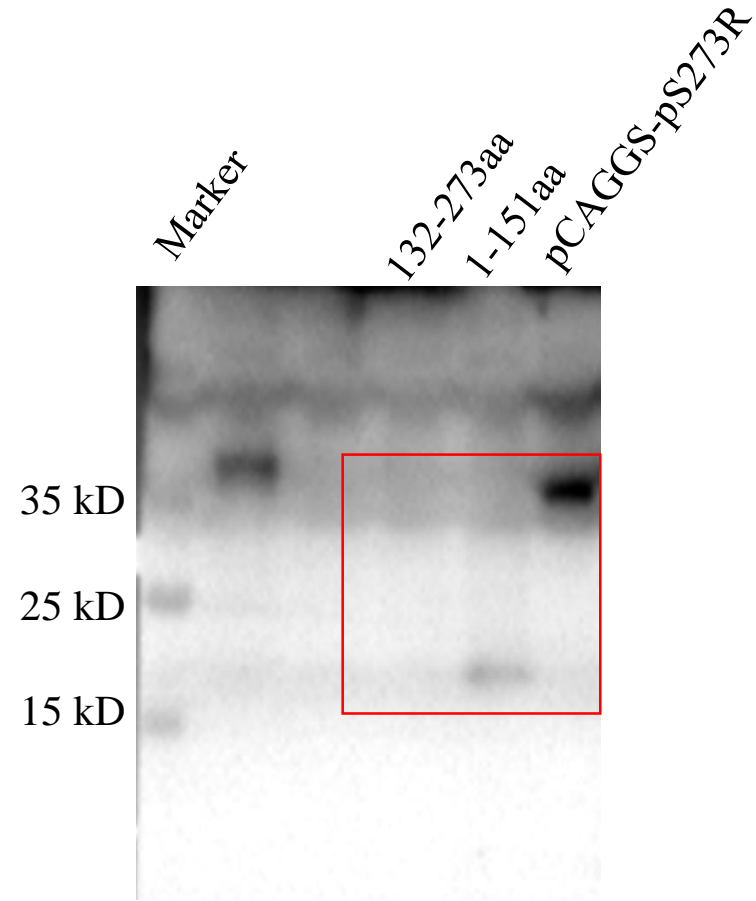

Figure S1

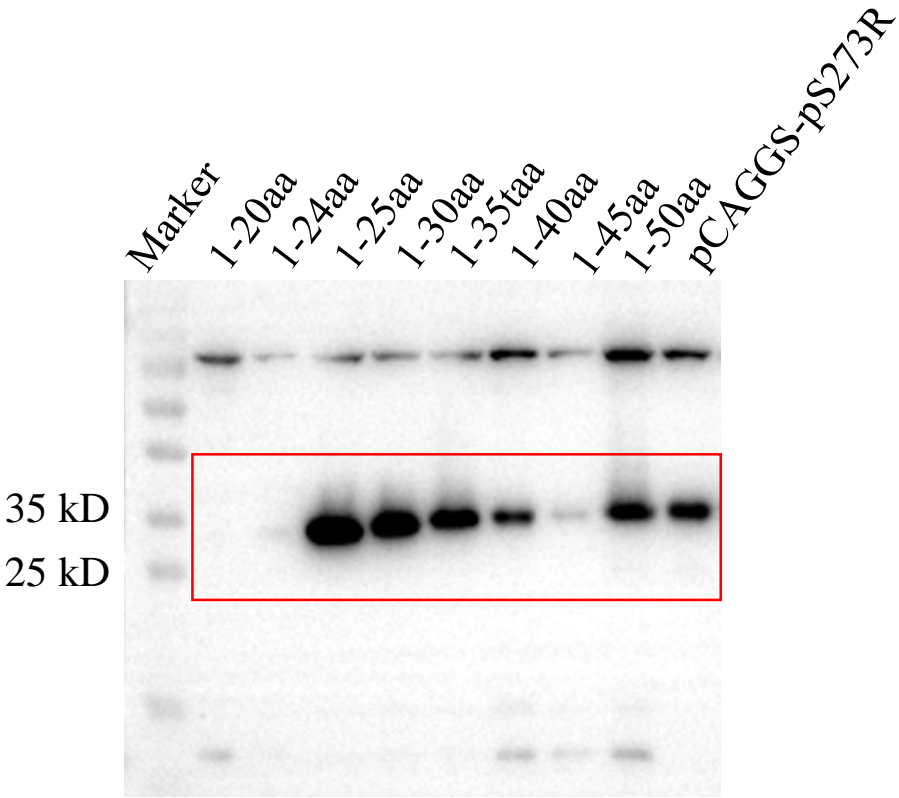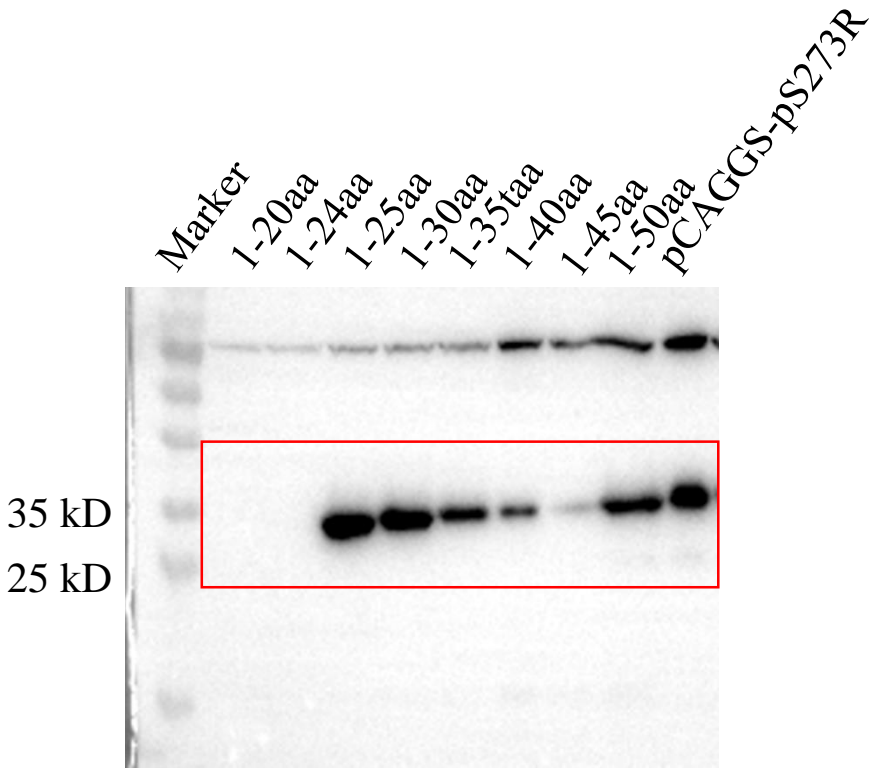

Supplement: Supplementary file 1 [file ijms-25-08906-s001.zip › Raw WB data.pdf]
